# Phytohormone Crosstalk of Cytokinin Biosynthesis and Signaling Family Genes in Moso Bamboo (*Phyllostachys edulis*)

**DOI:** 10.3390/ijms241310863

**Published:** 2023-06-29

**Authors:** Yucong Bai, Miaomiao Cai, Yuping Dou, Yali Xie, Huifang Zheng, Jian Gao

**Affiliations:** Key Laboratory of National Forestry and Grassland Administration, Beijing for Bamboo & Rattan Science and Technology, International Center for Bamboo and Rattan, Beijing 100102, China; bai.yucong@icbr.ac.cn (Y.B.); cmm@icbr.ac.cn (M.C.); douyuping@icbr.ac.cn (Y.D.); xieyali@icbr.ac.cn (Y.X.); zhenghuifang@icbr.ac.cn (H.Z.)

**Keywords:** cytokinin, pathway, *Phyllostachys edulis*, shoot growth

## Abstract

Cytokinin is widely involved in the regulation of plant growth, but its pathway-related genes have not been reported in Moso bamboo. In this study, a total of 129 candidate sequences were identified by bioinformatic methods. These included 15 *IPT* family genes, 19 *LOG* family genes, 22 *HK* family genes, 11 *HP* family genes and 62 *RR* family genes. Phylogenetic analysis revealed that the cytokinin pathway was closely related to rice, and evolutionary pattern analysis found that most of the genes have syntenic relationship with rice-related genes. The Moso bamboo cytokinin pathway was evolutionarily conservative and mainly underwent purifying selection, and that gene family expansion was mainly due to whole-gene duplication events. Analysis of transcriptome data revealed a tissue-specific expression pattern of Moso bamboo cytokinin family genes, with auxin and gibberellin response patterns. Analysis of co-expression patterns at the developmental stages of Moso bamboo shoots revealed the existence of a phytohormone co-expression pattern centered on cytokinin signaling genes. The auxin signaling factor PheARF52 was identified by yeast one-hybrid assay as regulating the *PheRR3* gene through a P-box element in the *PheRR3* promoter region. Auxin and cytokinin signaling crosstalk to regulate Moso bamboo growth. Overall, we systematically identified and analyzed key gene families of the cytokinin pathway in Moso bamboo and obtained key factors for auxin and cytokinin crosstalk, laying the foundation for the study of hormone regulation in Moso bamboo.

## 1. Introduction

Cytokinin (CK) plays an important role in physiological and biochemical responses such as cell division and expansion, induction of organ differentiation, delayed senescence, stress response and plant morphogenesis [1,2,3]. Since cytokinin and auxin were identified as the two most important phytohormones that play a regulatory role in plant tissue culture, cytokinin research has gradually intensified [4,5]. In the last decade, the molecular regulation of cytokinin synthesis and signal transduction has been continuously elucidated in parallel with advances in biotechnology [6,7,8].

In higher plants, cytokinin is mainly found in the form of zeatin, which includes both cis- and trans-zeatin, and trans-zeatin is the main active component of naturally occurring cytokinin. Trans-zeatin, isolated from immature maize seeds in 1963, was the first cytokinin (t-zeatin) to be discovered and purified [9]. There are two main forms of trans-zeatin synthesis in plants, one is the tRNA pathway, which involves the synthesis of highly active trans-zeatin catalyzed by cis-trans isomerases using cis-zeatin released from the breakdown of tRNA as a substrate [10,11]. However, this pathway is inefficient and does not meet the large amount of cytokinin required by plants. The other is the de novo synthesis pathway, which refers mainly to the cytokinin synthesis pathway that relies on two key rate-limiting enzymes [12,13] (Figure S1). The first step in CK synthesis relies on adenosine phosphate-isopentenyltransferase (IPT) to catalyze the conversion of adenosine-5-phosphate (including AMP, ATP and ADP) and dimethylallyl-polyphosphate (DMAPP) or hydroxymethylbutenyl diphosphate (HMBDP) to the nucleic acid form of isopentenyl adenosine (N6-(Δ2-isopentenyl) adenine (iP) riboside, iPR). A total of nine members of the *IPT* gene family have been identified in Arabidopsis, of which *AtIPT2* and *AtIPT9* encode tRNA-isopentenyltransferases (tRNA synthesis pathway) [14,15]. A total of eight members of the *IPT* gene family have been identified in rice [16]. The nucleic acid form of iPR is catalyzed by CYP735A1 or CYP735A2 of the cytochrome oxidase P450 family to produce the nucleic acid form of tZR, which is finally converted to active CK by cytokinin phosphate ribose hydrolase (LOG) [17].

CK signaling in higher plants is a complex process based on a binary signaling system consisting of histidine kinase (HK), phosphotransfer protein (HP) and response regulators (RR) [18,19] (Figure S1). The histidine kinase (HK) protein family is mainly located in the cell membrane as CK receptors to receive active CK. In Arabidopsis, *AHK2*, *AHK3* and *AHK4* are mainly located in the endoplasmic reticulum membrane and have different distributions and different affinities for CK to meet the demand for CK in different parts of the plant [20,21,22]. During CK signaling, HK first phosphorylate themselves and then transfer phosphate groups directly or indirectly to conserved aspartic acids, regulating the activity of the ligand signaling region. There are six members of the *AHP* gene family in Arabidopsis, which are responsible for the transfer of phosphate groups from the cytoplasm to the response regulator (RR) in the nucleus [18]. There are three main types of response regulators (RRs), of which Type-A *RRs* are the major cytokinin-responsive genes, negatively regulated by cytokinin transcriptional induction. Type-B *RRs* are positively regulated transcription factors in the cytokinin signaling pathway, and can bind directly to target DNA sequences to activate the expression of target genes. Type-A *ARRs* also act as signaling regulators and repress signaling by Type-B *ARRs*. Type-C *RRs* are similar to type-A *RRs*, but their expression is not induced by cytokinins [23]. There are 22 members of the *RR* gene family in Arabidopsis, including 11 type-A *ARRs* and 11 type-B *ARRs* [24,25].

Moso bamboo (*Phyllostachys edulis* (Carrière) J. Houzeau, synonym *Phyllostachys heterocycla* (Carrière)) belong to the monophyletic BEP clade (Bambusoideae, Ehrhartoideae, Pooideae) in the grass family (Poaceae), which is the most widely cultivated and economically valuable bamboo species, with rapid growth and strong representation [26,27]. During the rapid growth period, bamboo shoots can grow at a rate of up to 1 m/d, reaching a maximum height of around 20 m in 45–60 d [28]. During the entire growth period, plant hormones such as auxin, cytokinin and gibberellin play a crucial role. Gibberellin and auxin directly influence the internode length in Moso bamboo, while the dynamic balance between the cytokinin and auxin content affects internode cell elongation [29,30]. Here, we systematically identified gene families critical for CK synthesis and signal transduction, and conducted a preliminary investigation into the regulation of cytokinin and auxin, laying the foundation for studying the involvement of CK in the regulation of Moso bamboo growth.

## 2. Results

### 2.1. Identification and Characterization of Key Family Genes Acting on the CK Pathway in Moso Bamboo

A comprehensive analysis of Moso bamboo CK pathway biosynthesis and signaling family genes was conducted, and a total of 129 candidate sequences were identified in the Moso bamboo genome (Table S1). These included 15 *IPT* family genes, 19 *LOG* family genes, 22 *HK* family genes, 11 *HP* family genes and 62 *RR* family genes. Next, the basic physicochemical properties of the proteins encoded by the CK pathway biosynthesis and signaling genes were systematically assessed, including amino acid number, molecular weight, isoelectric point, instability index, lipolysis index and hydrophilicity index. Comprehensive analysis showed that CK-related proteins ranged in length from 90 aa (PheRR53) to 1245 aa (PheHK5), with predicted molecular weights ranging from 10.18 kDa to 136.43 kDa and isoelectric points of 4.56 (PheHP7) to 10.76 (PheRR22). The protein instability index ranged between 25.47 (PheRR39) and 68.38 (PheAP9). The predicted protein lipolysis index ranged from 52.33 (PheRR20) to 111.17 (PheRR56), and the protein hydrophilicity index ranged from −1.055 (PheRR59) to 0.359 (PheHP6). The HP gene family had the lowest average number of amino acids (181 aa) and the *HK* gene family had the highest average number of amino acids (419 aa). The isoelectric point and instability index were similar within the same gene family, but there were significant differences between the families, with the largest differences in the properties of the *HP* and *RR* gene families. These results suggest that although there is a divergence in gene function within the same gene family, the differences between the different gene families are clear. The *HP* gene family has the fewest members and the shortest average gene length, suggesting a more conservative gene function, and the *RR* gene family has the most members, which may be related to the fact that phytohormone signaling is the main mode of phytohormone regulation.

### 2.2. Phylogenetic Analysis of CK Pathway-Related Family Genes in Moso Bamboo

**Figure 1:**
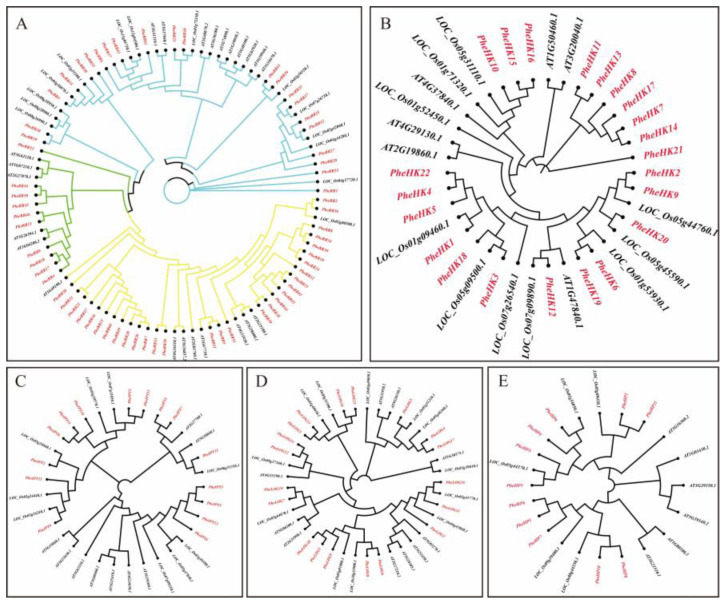
Phylogenetic tree of Moso bamboo cytokinin pathway family genes. (**A**–**E**) *PheRR* gene family, *PheHK* gene family, *PheIPT* gene family, *PheLOG* gene family and *PheHP* gene family, respectively. All phylogenetic trees were constructed from selected protein sequences of Moso bamboo, rice and Arabidopsis.

A phylogenetic tree was constructed by selecting Arabidopsis and rice CK pathway-related gene families together with Moso bamboo. In general, the CK pathway genes of Moso bamboo are more conserved in function, and Moso bamboo is more closely related to monocotyledonous rice, which is also in the grass family, and may potentially have more similar biological functions. Notably, there are 62 *RR* genes in Moso bamboo, including 22 Type-A *PheRR*, 30 Type-B *PheRR* and 10 Type-C *PheRR* genes. The expansion in the number of *PheRR* family members in Moso bamboo is much greater than in other gene families of the CK pathway, suggesting that *PheRR* genes may play a broader regulatory role in Moso bamboo, and also that there may be some redundancy in function (Figure 1).

### 2.3. Analysis of the Evolutionary Pattern of the CK Pathway in Moso Bamboo

**Figure 2:**
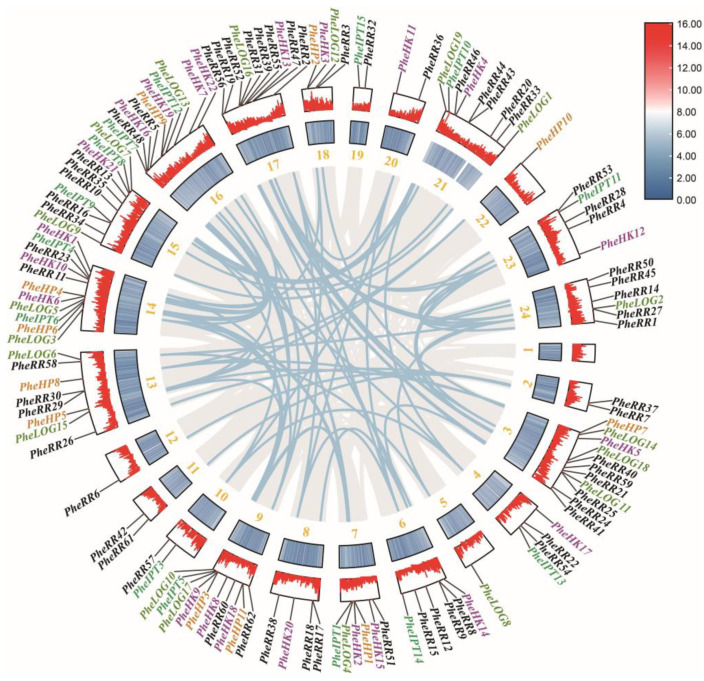
Chromosomal distribution and genome-wide replication analysis of genes in the Moso bamboo cytokinin pathway family. The blue lines represent syntenic gene pairs within the gene family. 1–24 represent chromosome 1—chromosome 24.

Analysis of the chromosomal distribution of the CK pathway family genes revealed that all chromosomes except Moso bamboo chromosome 1 had CK pathway genes distributed, with one gene distributed on chromosomes 5, 6 and 22 and two genes distributed above chromosomes 2, 10, 11, 19 and 20. CK pathway genes were mainly distributed on chromosomes 3, 9, 14, 15, 16 and 17, all of which contained more than ten related genes. Analysis of whole-genome duplication events within gene families revealed that a total of 85 whole-genome duplication events were obtained for 5 gene families, with only *PheHP5* and *PheHP8* above chromosome 13, and *PheRR50* and *PheRR45* on chromosome 24 having intrachromosomal tandem duplication. Genome-wide replication is the main driver of gene expansion in the CK pathway family in Moso bamboo (Figure 2).

**Figure 3:**
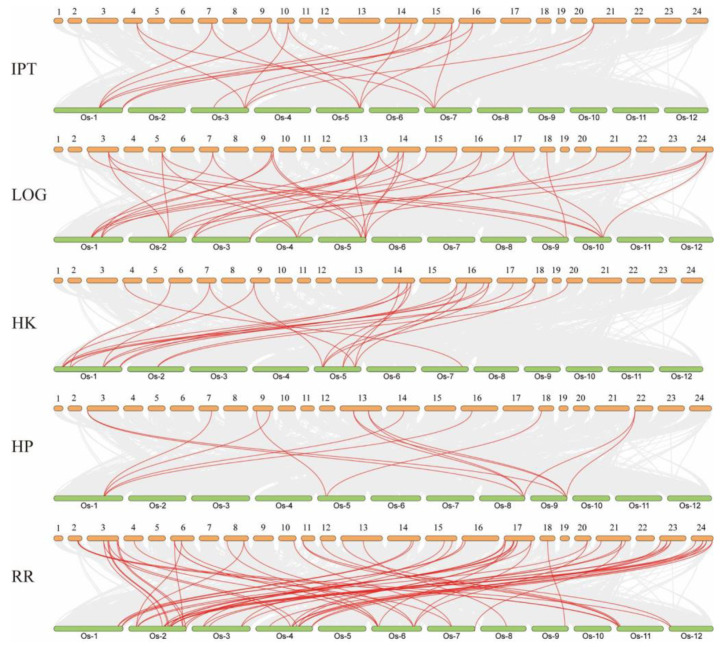
Syntenic analysis of genes in the Moso bamboo and rice cytokinin pathway families. Yellow represents Moso bamboo chromosomes, green represents rice chromosomes, and red lines represent homologous gene pairs between species. 1–24 represent chromosome 1—chromosome 24.

**Figure 4:**
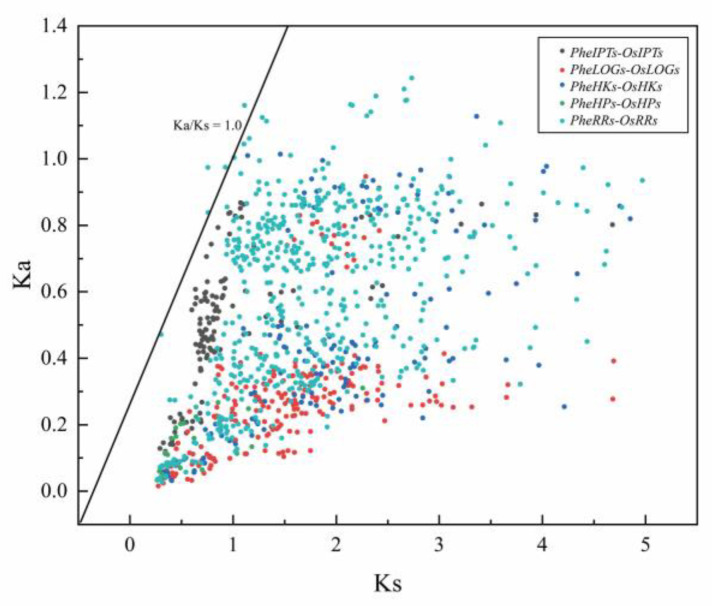
Selection pressure analysis of genes of the Moso bamboo cytokinin pathway family.

To further explore the evolutionary patterns of CK pathway genes, we selected closely related rice for syntenic analysis. Among them, there were 19 homologous genes in the *IPT* family, 35 pairs in *LOG*, 23 pairs in *HK*, 14 pairs in *HP* and 61 pairs in *RR* (Table S5). Most of the genes had syntenic relationships present, suggesting that the CK pathway may be more conserved in plants (Figure 3, Figure S2). Further selection pressure analysis revealed that the ratio of Ka/Ks < 1, indicating that the CK pathway is undergoing purifying selection and that the pathway is more conserved (Figure 4, Table S4).

### 2.4. Tissue-Specific Expression of CK Pathway-Related Family Genes

**Figure 5:**
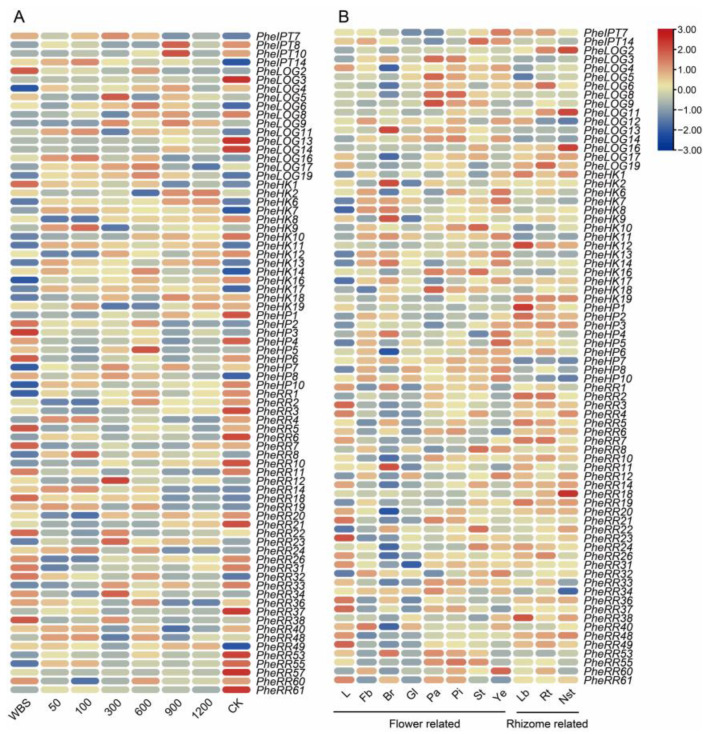
Tissue-specific expression analysis of Moso bamboo cytokinin pathway family genes. (**A**) Expression patterns of cytokinin family genes at different developmental stages of Moso bamboo shoots. WBS (winter bamboo shoots), 50–1200 represent bamboo shoot lengths (in cm), and CK represents bamboo with spreading leaves. (**B**) Expression patterns of different tissues of the Moso bamboo cytokinin pathway family genes. L (leaf), Fb (flower bud), Br (bract), Gl (glume), Pa (palea), Pi (pistil), St (stamen), Ye (young embryo). Lb (Lateral bud), Rt (Rhizome tip), Nst (New shoot tip).

Based on transcriptome data analysis, it was found that the expression trends of the CK pathway key gene family were significantly different at different stages of growth and development and in different tissues of Moso bamboo, and that the functions of different genes in the same family differed significantly, indicating that there were obvious tissue-specific expression patterns of CK pathway genes (Table S6). For the same gene, although there were still significant differences in expression in different tissues, the expression abundance was basically the same compared to other genes in the same family. Based on the expression analysis, it is clear that the CK pathway in Moso bamboo may rely on some of the key genes to play a major regulatory role, and that its mode of action is likely to be a synergistic regulation of several genes throughout the growth and developmental stages of the plant. For example, the main regulatory genes in CK synthesis may be *PheIPT7* and *PheIPT14*, and they play a major role in the below-ground part, which is also consistent with the earlier discovery of the main synthesis site of CK [31,32]. In Moso bamboo, the CK receptors are likely to be *PheHK7* and *PheHK17*, and the phosphate transporter protein is likely to be *PheHP8*, both of which are also highly expressed during the shoot growth stage, suggesting that cytokinin plays an important regulatory role during the shoot growth stage. At the same time, we found that most of the CK signaling genes were up-regulated during the bamboo maturation stage, while the synthesis and cascade signaling-related genes were expressed in the late shoot maturation stage, suggesting that other regulatory processes may exist between CK synthesis and regulatory effects (Figure 5).

### 2.5. Analysis of Auxin and Gibberellin Response of CK Pathway-Related Family Genes

**Figure 6:**
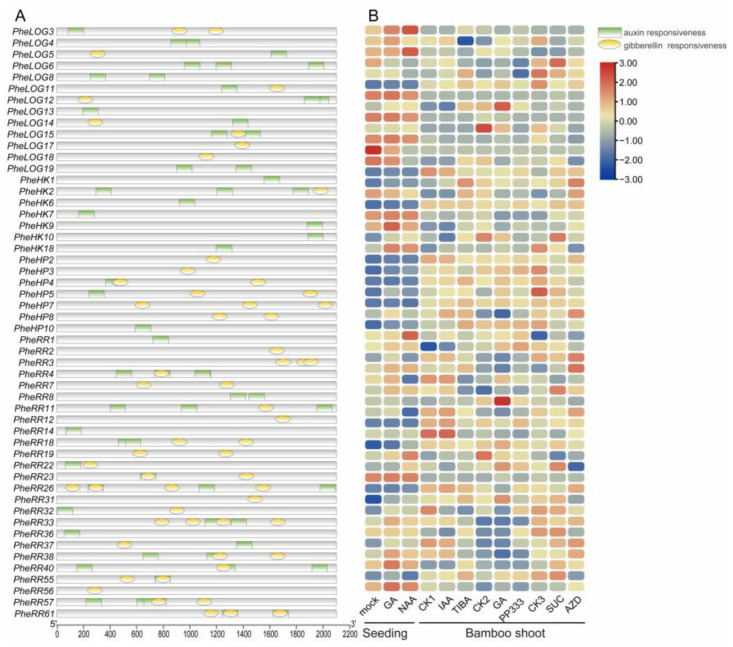
Analysis of the auxin and gibberellin response patterns of the Moso bamboo cytokinin family genes. (**A**) Auxin and gibberellin response elements in the promoter regions of the Moso bamboo cytokinin family genes (Table S3). (**B**) Expression patterns of the Moso bamboo cytokinin family genes under auxin and gibberellin treatments. Mock, GA, NAA represent the treatments of 1-month-old Moso bamboo seedlings with water, gibberellin (GA3), and acetic acid (NAA), respectively. Bamboo shoot treatments refer to 50 cm shoots treated with auxin (IAA), triiodobenzoic acid (TIBA), gibberellin (GA3) and paclobutrazol (PP333). The control was treated with water.

Cytokinin can positively promote plant growth and development, and their crosstalk with auxin and gibberellin has been widely reported [33,34,35]. We first performed a pooled analysis of the auxin and gibberellin response elements in the promoter regions of CK pathway genes and found that most gene promoter regions contained different amounts of auxin and gibberellin response elements. Next, we found that a large number of genes were induced or repressed in expression based on transcriptome data and analyses of Moso bamboo seedlings and field Moso bamboo shoots treated with auxin and gibberellin. In general, the response to auxin and gibberellin was stronger in seedlings than in Moso bamboo shoots, with auxin and gibberellin treatments inducing essentially the same expression trends, and most of the promoter regions of genes responding to auxin and gibberellin contained response elements. Specifically, in seedlings, *PheLOG5* could be heavily induced by auxin; *PheIPT7*, which may play a major regulatory role in synthesis, could be significantly repressed by gibberellin and auxin; while *PheIPT14* was insensitive to auxin but could be induced by gibberellin to up-regulate expression. In bamboo shoots, *PheLOG9* and *PheLOG13* could be induced by gibberellin for up-regulated expression, whereas *PheIPT7*, as in seedlings, was induced by auxin for down-regulated expression. For the *PheRR* gene family, the genes involved in regulation are not the same in seedlings as in bamboo shoots, and similarly, there are differences in the genes responding to gibberellin and auxin, with a large number of families assuming different regulatory roles in different modes (Figure 6). Overall, CK does respond to exogenous auxin and gibberellin in both seedlings and bamboo shoots, and may crosstalk with them to influence plant growth [36].

### 2.6. Analysis of Co-Expression Patterns of Key Gene Families of the Plant Hormone Pathway

**Figure 7:**
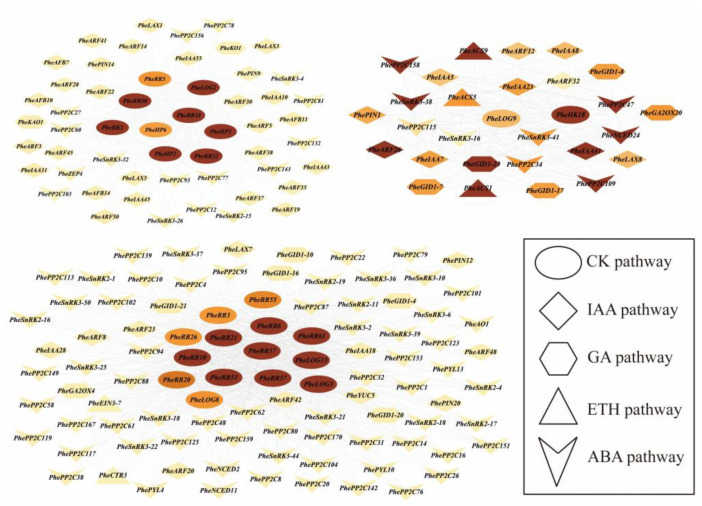
Analysis of co-expression patterns of genes related to the phytohormone signaling pathway in Moso bamboo shoots at different stages of development. Different shapes represent different phytohormones, with darker colors representing more co-expressed genes and higher Cytoscape software (V3.8.2) scores.

To further validate the role of phytohormone signaling crosstalk, we further identified five major classes of key genes of the phytohormone gene family, including auxin, gibberellin, ethylene, abscisic acid and cytokinin, and analyzed the co-expression pattern by the expression of all genes at different developmental stages of bamboo shoots (Table S2). The results showed that there was a co-expression network of phytohormone-related genes with CK as the core at different developmental stages of bamboo shoots. The core co-expressed genes were mainly the cytokinin signaling genes, *PheRR* genes, in addition to some *PheLOG* genes, *PheHK* genes and *PheHP* genes, which also played a central role. ABA, GA and IAA-related genes were the most frequently co-expressed genes (Figure 7, Table S7).

### 2.7. Validation of Upstream Regulators of PheRR3

**Figure 8:**
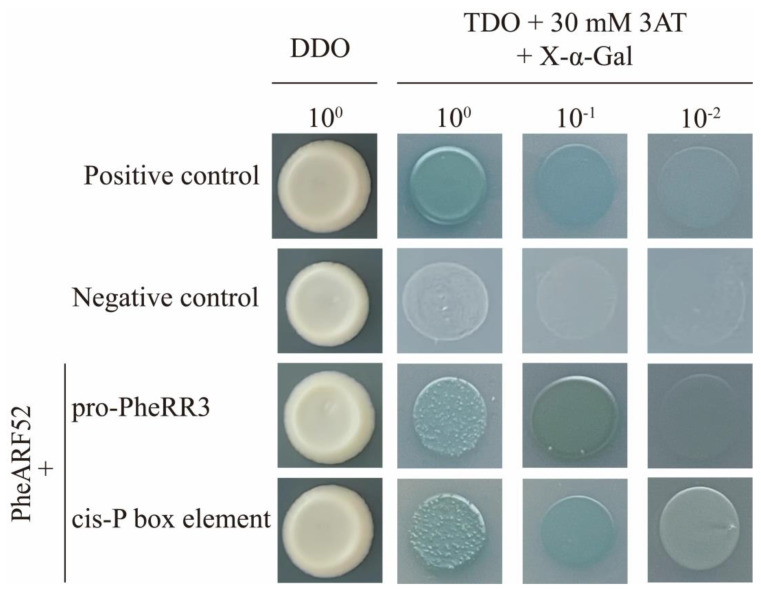
Analysis of upstream regulatory transcription factor of the *PheRR3* gene in Moso bamboo. Positive control is pGAD T7 53 + pHIS2 53 and negative control is pHIS2 53 + pGADT7 Rec2. pro-*PheRR3* represents the *PheRR3* promoter region and cis-P-box element represents the P-box element sequence (CCTTTTTG) repeated three times. All promoter sequences are linked to the pHIS2 vector and PheARF52 to the pGADT7 vector.

To further determine the regulatory role of cytokinin and other plant hormones, we cloned the promoter region of *PheRR3* (*PH02Gene05139.t1*) and used yeast one-hybrid assay to screen the yeast library of Moso bamboo shoots and obtain the upstream regulatory transcription factor PheARF52 (PH02Gene50607.t1). The CDS region of the *PheARF52* gene was re-cloned and the yeast one-hybrid assay was repeated to determine that PheARF52 can bind to the promoter of *PheRR3*. To verify the results, the promoter of *PheRR3* was analyzed and the relevant element was repeated three times for cloning and ligated into the yeast one-hybrid vector, and PheARF52 was found to bind the P-box element of the promoter region of *PheRR3* (Figure 8). Overall, PheARF52 can regulate *PheRR3* gene expression through the P-box element in the promoter region, enabling the crosstalk between auxin and cytokinin signaling.

## 3. Discussion

Cytokinin is closely associated with many regulatory processes of plant growth and development, including embryonic development, shoot differentiation, root growth, vascular bundle formation, flowering and leaf senescence [37,38]. As research continues to develop, members of genes related to the cytokinin pathway in model plants continue to be identified, the important role of cytokinin continues to be confirmed and the pathway becomes more clearly defined. Adenosine phosphate isopentenyltransferase (IPT) is an important rate-limiting enzyme in cytokinin synthesis. In Arabidopsis, using dimethylallyl diphosphate as a side chain donor, AtIPT4 isoprenylates ATP and ADP to form isoprenyl ATP and isoprenyl ADP. When the *AtIPT4* gene is overexpressed, stems can regenerate even in the absence of exogenous cytokinin [14]. Likewise, stem regeneration is usually only possible when cytokinins are applied to the healing tissue. This suggests that in the plant, the product of the gene catalyzes the biosynthesis of cytokinin. In Moso bamboo, a total of 15 *IPT* genes were identified, of which *PheIPT7* and *PheIPT14* were abundantly expressed in different parts of Moso bamboo shoots at all stages of growth, especially reaching the highest expression in the underground system (Figure 5), which is associated with the spatial- and temporal-specific expression of *IPT* genes that regulate cytokinin biosynthetic transport. In contrast, cytokinin phosphate ribose hydrolase (LOG) is clearly induced by auxin expression, which may play an important regulatory role in the crosstalk of auxin and cytokinin signals [39]. During the development of Moso bamboo shoots, cytokinin-signaling-related genes were abundantly expressed to facilitate the exercise of cytokinin’s important regulatory role, suggesting that cytokinin may be synergistically regulated with a large number of other phytohormones to facilitate the growth process during the growth of Moso bamboo. There are 62 *PheRR* genes in Moso bamboo, and excluding possible functional redundancy, the number of genes that play an important regulatory role may still be more than twice that of Arabidopsis, which may indicate that cytokinin plays a greater role than expected in Moso bamboo.

Through evolutionary tree construction we initially determined that Moso bamboo was more closely related to rice, and therefore selected rice and Moso bamboo for syntenic analysis, and found that most of the Moso bamboo genes had syntenic relationships related to rice-related genes. Further analysis by selection pressure determined that the cytokinin pathway was relatively evolutionarily conserved, and that the entire pathway was subject to purifying selection, and that gene family expansion was mainly due to genome-wide replication within the gene family, which is consistent with the conclusion that the phytohormone pathway genes are functionally conserved [40].

In Moso bamboo, previous studies have found that the phytohormone content is not the same in different parts of the same internode in bamboo shoots, and that the content of auxin, gibberellin and cytokinin is much higher at the base of the internode than at the upper part of the internode [41]. At the same time, we have identified the role of auxin and gibberellin in the regulation of the internode length and plant height, and the possible involvement of cytokinin in the regulation of elongation in the internode elongation zone of Moso bamboo, but the molecular mechanisms involved are not yet clear [29,30,42]. In order to further analyze the integration of phytohormone signaling in Moso bamboo, we found that most of the genes containing the corresponding regulatory elements in the promoter region could respond to the corresponding phytohormones based on the available transcriptome data, and further determined the phytohormone co-expression pattern during the developmental stage of bamboo shoots. This is more consistent with the results of previous studies on the synergistic regulation of potato growth by auxin and cytokinin [43]. We therefore further validated the results by yeast one-hybrid assay, which showed that the auxin signaling factor ARF could bind the P-box element in the promoter region of the cytokinin signaling gene, that there was signaling crosstalk between the auxin signal and the cytokinin signal, and that cytokinin may be involved in the regulation of Moso bamboo through auxin. The effect of cytokinin on the regulation of plant height is likely to be mediated by auxin [36,39].

## 4. Materials and Methods

### 4.1. Identification and Physicochemical Property Analysis of Phytohormone Pathway Genes in Moso Bamboo

The phytohormone pathway protein sequences of Arabidopsis and rice were retrieved from the Arabidopsis genome database (http://www.arabidopsis.org/, accessed on 23 May 2023) and the rice genome database (http://rice.plantbiology.msu.edu/index.shtml, accessed on 23 May 2023), and blast to the local database based on Moso bamboo genomic data to retrieve members of the Moso bamboo phytohormone pathway gene family. conserved structural domain analysis from the NCBI database (https://www.ncbi.nlm.nih.gov/Structure/cdd/wrpsb.cgi, accessed on 23 May 2023) was used to screen the conserved structural domains of the Moso bamboo phytohormone pathway protein sequences for identification. The number of amino acids, molecular weight, theoretical isoelectric point, instability coefficient, hydrophilicity index and lipolysis index of the CK pathway gene family of Moso bamboo were obtained using the online tool ExPASY (https://www.expasy.org/tools, accessed on 23 May 2023).

### 4.2. Phylogenetic Tree Construction and Evolutionary Pattern Analysis

MEGA6.0 software was used to compare and analyze the full-length multiple sequences of Arabidopsis, rice and Moso bamboo proteins identified in this study [44]. The phylogenetic tree was constructed by using the no-rooted neighbor-joining method and 1000 replicates were performed for the bootstrap analysis. Chromosomal distribution of CK pathway-related genes, genome replication analysis, interspecies syntenic analysis and selection pressure analysis were performed using TBtools software, (V1.126) including Advanced Circos, One Step MCScanX and Simple Ka/KS Calculator programs [45].

### 4.3. Promoter Analysis and Transcriptome Data Acquisition

The 2000 bp sequence upstream of gene was extracted as the promoter region by TBtools software, all promoters were aggregated and cis-acting element prediction was performed through the PlantCARE cis-acting element website (http://bioinformatics.psb.ugent.be/webtools/plantcare/html/, accessed on 23 May 2023). TBtools software was used for visualization. Transcriptome data were obtained from the NCBI GEO database (https://www.ncbi.nlm.nih.gov/geo/, accessed on 23 May 2023), accesss numbers are GSE104596, GSE100172, GSE90517 [28,29,46,47,48]. The RPKM values of gene expression were used to analyze the expression levels of CK pathway genes. For statistical purposes, the log 2 of each expression was taken and gene expression heat maps were plotted using TBtools.

### 4.4. Co-Expression Analysis of Genes Related to the CK Pathway

Expression of the identified auxin, cytokinin, gibberellin, abscisic acid and ethylene related genes were tuned to different developmental stages of Moso bamboo. Co-expression patterns were performed by WGCNA package in R v1.29 with weights set to 6 and minimum module set to 30, and finally visualized by Cytoscape software [49,50].

### 4.5. Yeast One-Hybrid Assay

The promoter region of *PheRR3* (*PH02Gene05139.t1*) was cloned and ligated to the pHIS2 vector, the yeast library retained in the laboratory was screened, the full-length CDS sequence of the obtained *PheARF52* (*PH02Gene50607.t1*) target gene was ligated to the pGADT7 vector, and the regulatory element of the promoter region of *PheRR3* was extracted and repeated three times for cloning. The specific regulatory elements were identified by yeast one-hybrid assay. Yeast one-hybrid assay was carried out according to the clontech yeast one-hybrid system instructions (630491).

## Data Availability

All the data to support the study results in this paper are within its Supplementary Information.

## Supplementary Materials

The following supporting information can be downloaded at https://www.mdpi.com/article/10.3390/ijms241310863/s1.
