# Supplementary material for: Phytohormone Crosstalk of Cytokinin Biosynthesis and Signaling Family Genes in Moso Bamboo (Phyllostachys edulis)
Source: Int J Mol Sci. 2023 Jun 29;24(13):10863. doi: 10.3390/ijms241310863 (PMC10341911; doi:10.3390/ijms241310863)
Supplement: Supplementary file 1 [file ijms-24-10863-s001.zip › Supplementary Captions.pdf]

Figure S1. Pattern of gene families related to the cytokinin pathway.

Figure S2. Syntenic analysis of genes in the Moso bamboo and rice cytokinin pathway families.

Table S1. Identification of CK pathway gene family and physicochemical property analysis of Moso bamboo.

Table S2. Information on the identified phytohormone gene family members in Moso bamboo.

Table S3. Analysis of the cis-acting elements of the CK pathway gene family promoter in Moso bamboo.

Table S4. Specific information on Moso bamboo and rice selection pressure analysis.

Table S5. Genetic information on the syntenic relationships of Moso bamboo and rice.

Table S6. Expression of the CK pathway genes in Moso bamboo.

Table S7. Co-expressed gene information of phytohormone-related genes in Moso bamboo.
