# Supplementary figures and images for: Phytohormone Crosstalk of Cytokinin Biosynthesis and Signaling Family Genes in Moso Bamboo (Phyllostachys edulis)
Source: Int J Mol Sci. 2023 Jun 29;24(13):10863. doi: 10.3390/ijms241310863 (PMC10341911; doi:10.3390/ijms241310863)

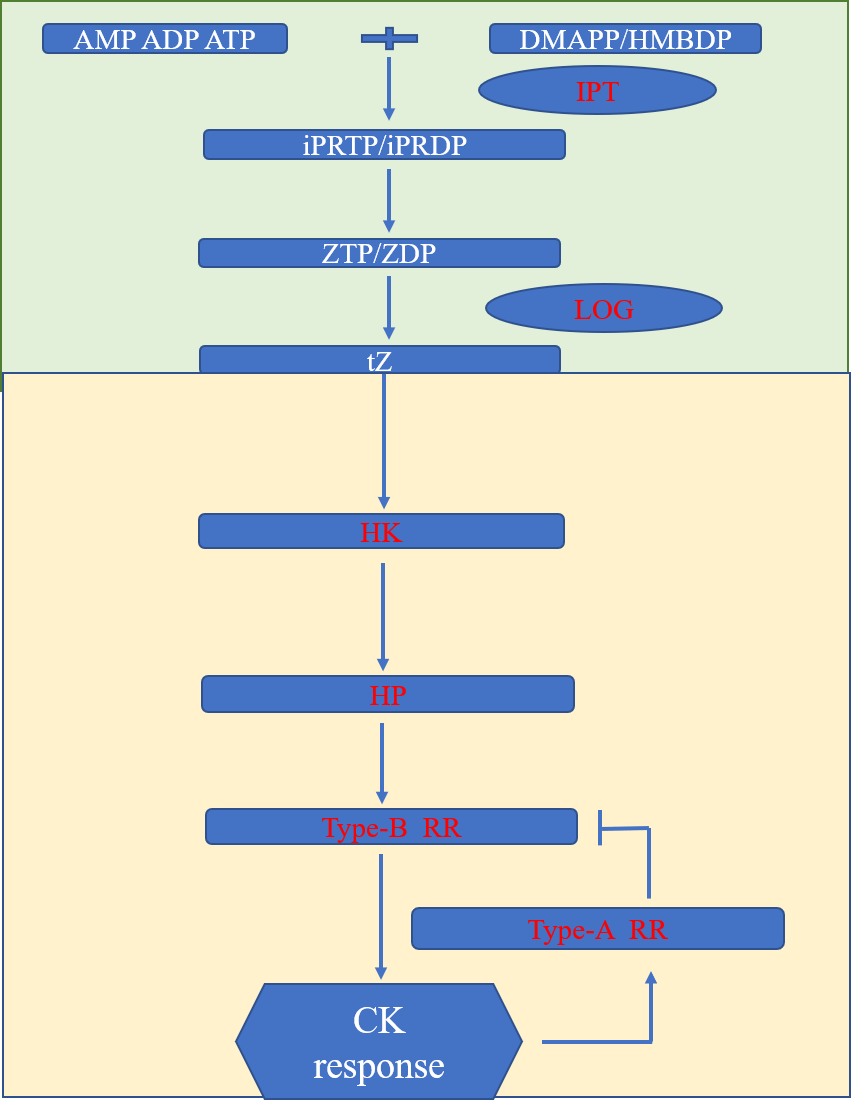

Supplement: Supplementary file 1 [file ijms-24-10863-s001.zip › FIGS1.tif]

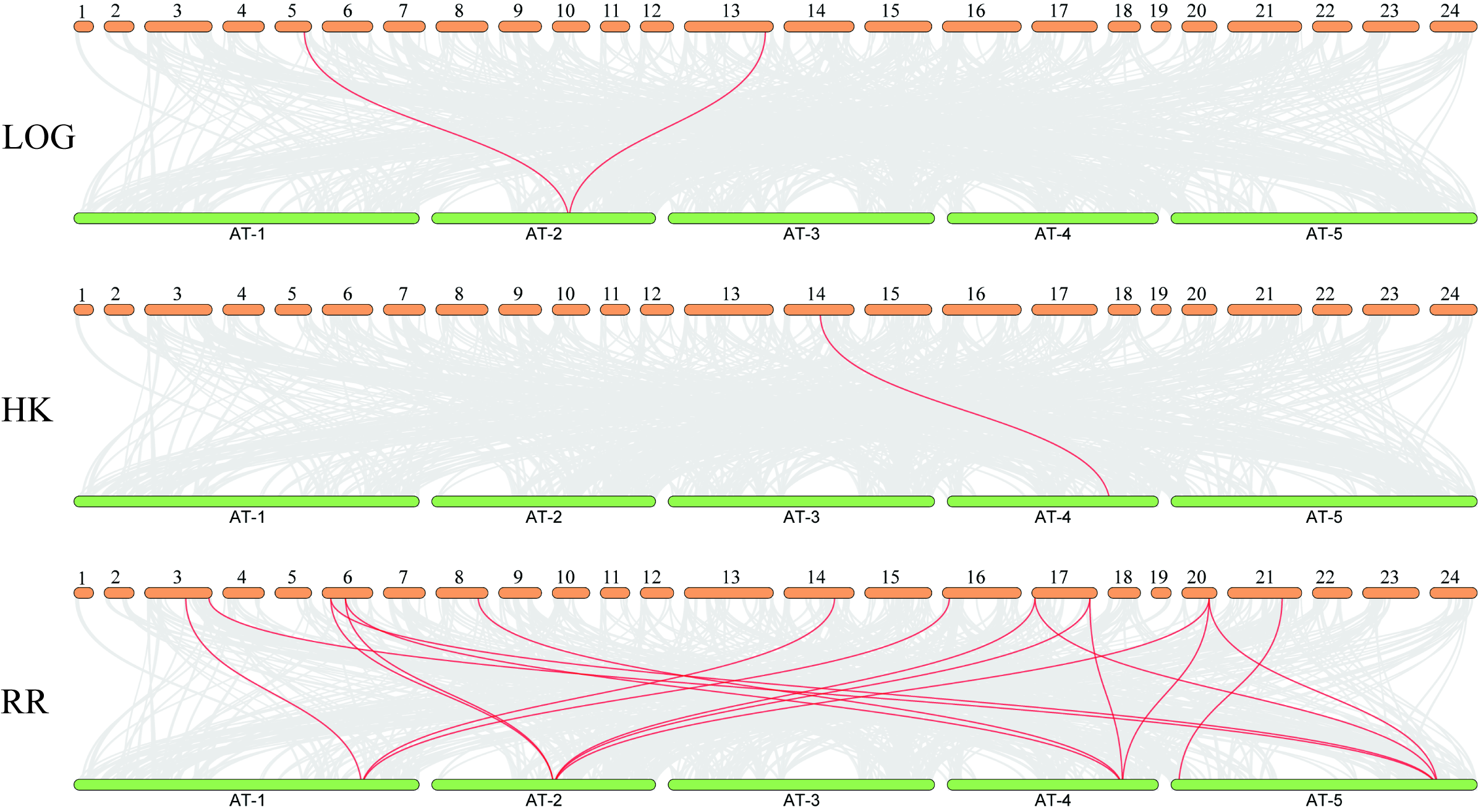

Supplement: Supplementary file 1 [file ijms-24-10863-s001.zip › FIGS2.tif]
